# Supplementary figures and images for: Mastitis risk effect on the economic consequences of paratuberculosis control in dairy cattle: A stochastic modeling study
Source: PLoS One. 2019 Sep 26;14(9):e0217888. doi: 10.1371/journal.pone.0217888 (PMC6762148; doi:10.1371/journal.pone.0217888)

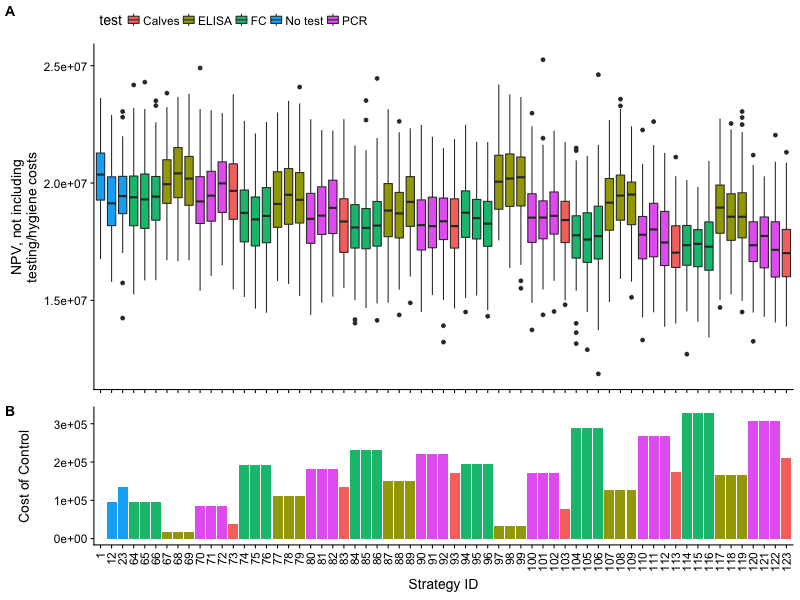

Supplement: S1 Fig — (TIFF) [file pone.0217888.s001.tiff]
