# Supplementary material for: Mastitis risk effect on the economic consequences of paratuberculosis control in dairy cattle: A stochastic modeling study
Source: PLoS One. 2019 Sep 26;14(9):e0217888. doi: 10.1371/journal.pone.0217888 (PMC6762148; doi:10.1371/journal.pone.0217888)
Supplement: S1 Table — (DOCX) [file pone.0217888.s002.docx]

**S1 Table.** Events, changes, and rates used for simulation via Gillespie’s direct algorithm^a^

| Event | Change | Rate |
| --- | --- | --- |
| Death of a susceptible calf | S_1_→S_1_-1 | μ_1_S_1_ |
| Death of a transiently shedding calf | T_1_→T_1_-1 | μ_1_T_1_ |
| Death of a susceptible heifer | S_2_→S_2_-1 | μ_2_S_2_ |
| Death of a transiently shedding heifer | T_2_→T_2_-1 | μ_2_T_2_ |
| Death of a latent heifer | E_2_→E_2_-1 | μ_2_E_2_ |
| Death of a susceptible adult | S_3_→S_3_-1 | μ_3_S_3_ |
| Death of a latent progressing adult | E_P_→E_P_-1 | μ_3_E_P_ |
| Death of a low-shedding progressing adult | L_P_→L_P_-1 | μ_3_L_P_ |
| Death of a high-shedding adult | H→H-1 | μ_3_H |
| Death of a latent non-progressing adult | E_N_→E_N_-1 | μ_3_E_N_ |
| Death of a low-shedding non-progressing adult | L_N_→L_N_-1 | μ_3_L_N_ |
| Aging of a susceptible calf | S_1_→S_1_-1, S_2_→S_2_+1 | ρ_1_S_1_ |
| Aging of a transiently shedding calf | T_1_→T_1_-1, T_2_→T_2_+1 | ρ_1_T_1_ |
| Aging of a susceptible heifer | S_2_→S_2_-1, S_3_→S_3_-1 | ρ_2_S_2_ |
| Aging of a latent heifer to progressing | E_2_→E_2_-1, E_P_→E_P_+1 | ηρ_2_E_2_ |
| Aging of a latent heifer to non-progressing | E_2_→E_2_-1, E_N_→E_N_+1 | (1-η)ρ_2_E_2_ |
| Transition of transiently shedding heifer to latency | T_2_→T_2_-1, E_2_→E_2_+1 | φT_2_ |
| Transition of latent progressor to low-shedding | E_P_→E_P_-1, L_P_→L_P+_1 | σ_H_E_P_ |
| Transition of low-shedding progressor to high-shedding | L_P_→L_P_-1, H→H+1 | νL_P_ |
| Transition of latent non-progressor to low-shedding | E_N_→E_N_-1, L_N_→L_N_+1 | σ_L_E_N_ |
| Birth of a susceptible calf | S_1_→S_1_+1 | μ_b_(1-γ) |
| Birth of a transiently shedding calf | T_1_→T_1_+1 | μ_b_γ |
| Infection of a susceptible calf | S_1_→S_1_-1, T_1_→T_1_+1 | λ_1_ |
| Infection of a susceptible heifer | S_2_→S_2_-1, T_2_→T_2_+1 | λ_2_ |
| Infection of a susceptible adult | S_3_→S_3_-1, E_N_→E_N_+1 | λ_3_ |
| Mastitis in a susceptible adult | none | ψ |
| Mastitis in an infected adult | none | ξ_I_ψ |

^a^Gillespie’s direct algorithm is applied in two steps. First, the time to the next event is found by drawing from an exponential distribution whose rate is the sum of the rates of all possible events. Second, the identity of the next event is found by drawing the event at random from a weighted multinomial distribution representing all possible events, where the weight of a given even is its rate.
