# Supplementary material for: Mastitis risk effect on the economic consequences of paratuberculosis control in dairy cattle: A stochastic modeling study
Source: PLoS One. 2019 Sep 26;14(9):e0217888. doi: 10.1371/journal.pone.0217888 (PMC6762148; doi:10.1371/journal.pone.0217888)
Supplement: S2 Table — (DOCX) [file pone.0217888.s003.docx]

**S2 Table.** ELISA-based strategies and their NPV distribution and number of dominated strategies for each mastitis scenario and herd type.

|  | Mastitis Association (MA) | | | | | | | |
| --- | --- | --- | --- | --- | --- | --- | --- | --- |
| Herd Size | 100 head | | | | 1000 head | | | |
| Prevalence | 7% | | 20% | | 7% | | 20% | |
|  | median  (range) | SOSD | median (range) | SOSD | median (range) | SOSD | median (range) | SOSD |
| No control | 1.92  (0.41,3.54) | 0 | 1.95 (1.13,3.03) | 8 | 20.64 (17.49,24.64) | 12 | 20.36 (16.76,23.63) | 5 |
| Annual ELISA, cull all | 1.84  (0.57,3.24) | 0 | 1.77 (0.59,3.07) | 0 | 18.64 (15.04,22.36) | 0 | 17.97 (13.44,21.79) | 0 |
| Annual ELISA, cull high | 1.96  (0.83,3.06) | 6 | 1.94 (0.81,3.24) | 2 | 20.33 (16.04,24.66) | 1 | 20.25 (15.97,24.97) | 2 |
| Annual ELISA, cull after 2 | 1.97  (0.89,3.18) | 5 | 2 (0.51,2.91) | 0 | 20.54 (16.52,23.02) | 9 | 19.85 (17.14,23.82) | 4 |
| Biannual ELISA, cull all | 1.9  (0.65,3.65) | 0 | 1.86 (0.54,3.63) | 0 | 20.37 (14.85,23.78) | 0 | 19.95 (16.74,24.52) | 2 |
| Biannual ELISA, cull high | 2.04  (0.5,3.26) | 1 | 1.97 (0.72,3.04) | 3 | 19.86 (16.45,24.12) | 1 | 19.77 (16.18,23.43) | 1 |
| Biannual ELISA, cull after 2 | 1.87  (0.73,3.4) | 1 | 2.04 (0.79,3.1) | 7 | 20.16 (15.39,23.8) | 1 | 19.69 (16.53,22.88) | 1 |
| Cont. annual ELISA, cull all | 1.93  (0.75,2.84) | 0 | 1.95 (0.65,3.52) | 2 | 20.66 (16.44,23.55) | 9 | 19.94 (16.69,23.82) | 6 |
| Cont. annual ELISA, cull high | 1.96  (0.83,3.31) | 3 | 2.03 (0.77,3.17) | 5 | 20.35 (16.24,24.41) | 2 | 20.4 (16.65,23.66) | 6 |
| Cont. annual ELISA, cull after 2 | 1.97  (0.57,3.22) | 0 | 1.93 (0.91,3.07) | 3 | 20.58 (14.49,24.55) | 0 | 20.17 (16.69,23.79) | 4 |
| Cont. biannual ELISA, cull all | 2.03  (0.89,3.43) | 9 | 1.95 (0.84,2.92) | 3 | 20.28 (16.33,23.8) | 3 | 20.02 (17.53,24.17) | 4 |
| Cont. biannual ELISA, cull high | 1.89  (0.99,3.37) | 7 | 1.84 (0.59,2.73) | 1 | 20.23 (16.11,24.3) | 1 | 20.16 (16.35,23.74) | 3 |
| Cont. biannual ELISA, cull after 2 | 1.95  (0.85,3.12) | 3 | 1.96 (0.64,3.3) | 4 | 20.54 (16.67,23.37) | 9 | 20.21 (15.48,23.64) | 1 |

|  | No Mastitis Association (NMA) | | | | | | | |
| --- | --- | --- | --- | --- | --- | --- | --- | --- |
| Herd Size | 100 head | | | | 1000 head | | | |
| Prevalence | 7% | | 20% | | 7% | | 20% | |
|  | median (range) | SOSD | median (range) | SOSD | median (range) | SOSD | median (range) | SOSD |
| No control | 1.98 (0.53,2.85) | 1 | 1.96 (0.98,2.92) | 8 | 20.45 (15.99,24.65) | 1 | 20.55 (16.99,23.85) | 11 |
| Annual ELISA, cull all | 1.75 (0.59,2.97) | 0 | 1.81 (0.45,3.28) | 0 | 18.69 (14.13,22.25) | 0 | 18.29 (12.74,22.02) | 0 |
| Annual ELISA, cull high | 2.01 (0.86,2.76) | 8 | 1.93 (1.07,3.21) | 12 | 20.31 (15.74,25.41) | 1 | 20.15 (15.21,23.74) | 1 |
| Annual ELISA, cull after 2 | 2 (0.81,3.29) | 7 | 1.89 (0.66,3.12) | 3 | 20.49 (16.26,24.22) | 5 | 19.75 (15.52,24.48) | 1 |
| Biannual ELISA, cull all | 1.93 (0.52,3.15) | 1 | 2.02 (0.8,3.33) | 7 | 20.19 (15.63,23.64) | 1 | 19.96 (16.92,23.39) | 3 |
| Biannual ELISA, cull high | 2.04 (0.95,2.95) | 11 | 1.86 (0.36,3.28) | 1 | 20.27 (17.32,24.09) | 7 | 19.84 (16.33,23.13) | 1 |
| Biannual ELISA, cull after 2 | 2 (0.94,3.28) | 8 | 1.92 (0.67,3.05) | 3 | 20.06 (16.38,23.98) | 3 | 20.43 (16.35,23.76) | 5 |
| Cont. annual ELISA, cull all | 2 (1.11,3.31) | 8 | 1.96 (0.8,3.36) | 7 | 20.11 (16.38,24.66) | 4 | 20.13 (15.92,24.22) | 2 |
| Cont. annual ELISA, cull high | 2 (0.8,3.29) | 4 | 1.96 (0.21,2.87) | 0 | 20.49 (15.44,23.83) | 1 | 20.56 (16.06,23.69) | 5 |
| Cont. annual ELISA, cull after 2 | 1.96 (0.86,3.26) | 5 | 1.95 (0.4,3.06) | 0 | 20.68 (16.07,24.46) | 5 | 20.46 (16.76,24.22) | 6 |
| Cont. biannual ELISA, cull all | 1.87 (0.39,3.21) | 0 | 1.96 (0.7,3.13) | 4 | 19.78 (15.89,23.98) | 1 | 19.89 (16.52,23.81) | 1 |
| Cont. biannual ELISA, cull high | 1.9 (0.59,3.32) | 3 | 1.93 (0.77,3.2) | 7 | 19.88 (16.96,25.16) | 2 | 20.02 (16.17,24.6) | 1 |
| Cont. biannual ELISA, cull after 2 | 2.01 (0.78,3.15) | 3 | 1.78 (0.86,2.99) | 1 | 20.33 (17.3,23.59) | 8 | 20.2 (15.84,24.22) | 3 |

|  | No Mastitis (NM) | | | | | | | | |
| --- | --- | --- | --- | --- | --- | --- | --- | --- | --- |
| Herd Size | 100 head | | | | | 1000 head | | | |
| Prevalence | 7% | | 20% | | 7% | | | 20% | |
|  | median (range) | SOSD | median (range) | SOSD | median (range) | | SOSD | median (range) | SOSD |
| No control | 2.12 (1.01,3.22) | 6 | 2.08 (0.86,3.33) | 7 | 21.23 (17.98,25.43) | | 11 | 21.08 (14.59,26.41) | 1 |
| Annual ELISA, cull all | 1.86 (0.83,3.1) | 0 | 1.86 (0.16,2.81) | 0 | 18.9 (14.06,25.37) | | 0 | 18.4 (10.95,23.13) | 0 |
| Annual ELISA, cull high | 1.99 (0.63,3.25) | 0 | 1.92 (0.78,3.49) | 4 | 20.61 (15.63,24.04) | | 1 | 20.5 (16.07,24.86) | 2 |
| Annual ELISA, cull after 2 | 1.87 (0.88,3.28) | 1 | 1.97 (0.99,3.41) | 2 | 20.74 (17.08,25.14) | | 3 | 20.28 (17.22,23.36) | 3 |
| Biannual ELISA, cull all | 1.92 (0.58,3.19) | 0 | 1.97 (0.83,3.7) | 7 | 20.85 (16.33,24.97) | | 2 | 20.58 (15.72,24.79) | 1 |
| Biannual ELISA, cull high | 2.04 (0.73,3.24) | 1 | 2.01 (0.74,3.16) | 1 | 21.07 (16.37,23.82) | | 2 | 20.43 (16.59,23.12) | 2 |
| Biannual ELISA, cull after 2 | 2.06 (0.63,3.2) | 0 | 1.93 (0.43,3.18) | 1 | 21.22 (16.87,25.16) | | 7 | 20.11 (16.33,24.7) | 2 |
| Cont. annual ELISA, cull all | 2.01 (0.86,2.95) | 2 | 1.88 (0.89,2.76) | 1 | 20.93 (15.28,24.96) | | 1 | 20.45 (16.19,24.51) | 3 |
| Cont. annual ELISA, cull high | 2.01 (1.05,3.13) | 3 | 2.03 (0.79,3.12) | 6 | 21.17 (16.21,24.83) | | 3 | 20.57 (17.43,24.02) | 8 |
| Cont. annual ELISA, cull after 2 | 2.05 (1.03,3.5) | 8 | 1.99 (0.81,2.96) | 2 | 20.87 (17.02,25.59) | | 6 | 20.55 (16.95,24.85) | 6 |
| Cont. biannual ELISA, cull all | 1.97 (0.93,2.91) | 3 | 2.01 (0.45,3.07) | 1 | 20.72 (17.24,25.31) | | 3 | 20.14 (15.9,23.58) | 1 |
| Cont. biannual ELISA, cull high | 2.02 (1.15,3.18) | 9 | 1.83 (0.7,3.32) | 1 | 20.69 (15.79,24.46) | | 1 | 20.41 (17.62,24.56) | 6 |
| Cont. biannual ELISA, cull after 2 | 2.06 (1.18,3.73) | 9 | 2.03 (0.32,3) | 0 | 20.78 (17.19,23.02) | | 3 | 20.05 (16.7,23.99) | 1 |

SOSD is the proportion of strategies second-order dominated
