# Supplementary material for: Mastitis risk effect on the economic consequences of paratuberculosis control in dairy cattle: A stochastic modeling study
Source: PLoS One. 2019 Sep 26;14(9):e0217888. doi: 10.1371/journal.pone.0217888 (PMC6762148; doi:10.1371/journal.pone.0217888)
Supplement: S3 Table — (DOCX) [file pone.0217888.s004.docx]

**S3 Table**. Discounted cost of implementing different possible paratuberculosis controls, not including culling and replacement costs, over a 5 year period in a 1,000-head dairy herd.

| Control | Discounted Cost ($) |
| --- | --- |
| ELISA Annual | 16148 |
| FC Annual | 96890 |
| PCR Annual | 86124 |
| Calves Annual | 39027 |
| ELISA Biannual | 32297 |
| FC Biannual | 193780 |
| PCR Biannual | 172249 |
| Calves Biannual | 78055 |
| Moderate Hygiene | 95652 |
| High Hygiene | 133600 |
